# The effects of micro-implant assisted rapid palatal expansion (MARPE) on the nasomaxillary complex—a finite element method (FEM) analysis

**DOI:** 10.1186/s40510-014-0052-y

**Published:** 2014-08-29

**Authors:** Matt MacGinnis, Howard Chu, George Youssef, Kimberley W Wu, Andre Wilson Machado, Won Moon

**Affiliations:** UCLA Section of Orthodontics, UCLA School of Dentistry, 10833 Le Conte Avenue, CHS - Box 951668, Los Angeles, CA 90095-1668 USA; Mechanical Engineering Department, California State University, Northridge, 18111 Nordhoff Street, Northridge, CA 91330 USA; Section of Orthodontics, Federal University of Bahia, Salvador, Bahia 40110912 Brazil

**Keywords:** Finite element method (FEM), Rapid palatal expansion (RPE), Micro-implant assisted rapid palatal expansion (MARPE), Nasomaxillary sutures

## Abstract

**Background:**

Orthodontic palatal expansion appliances have been widely used with satisfactory and, most often, predictable clinical results. Recently, clinicians have successfully utilized micro-implants with palatal expander designs to work as anchors to the palate to achieve more efficient skeletal expansion and to decrease undesired dental effects. The purpose of the study was to use finite element method (FEM) to determine the stress distribution and displacement within the craniofacial complex when simulated conventional and micro-implant-assisted rapid palatal expansion (MARPE) expansion forces are applied to the maxilla. The simulated stress distribution produced within the palate and maxillary buttresses in addition to the displacement and rotation of the maxilla could then be analyzed to determine if micro-implants aid in skeletal expansion.

**Methods:**

A three-dimensional (3D) mesh model of the cranium with associated maxillary sutures was developed using computed tomography (CT) images and Mimics modeling software. To compare transverse expansion stresses in rapid palatal expansion (RPE) and MARPE, expansion forces were distributed to differing points on the maxilla and evaluated with ANSYS simulation software.

**Results:**

The stresses distributed from forces applied to the maxillary teeth are distributed mainly along the trajectories of the three maxillary buttresses. In comparison, the MARPE showed tension and compression directed to the palate, while showing less rotation, and tipping of the maxillary complex. In addition, the conventional hyrax displayed a rotation of the maxilla around the teeth as opposed to the midpalatal suture of the MARPE. This data suggests that the MARPE causes the maxilla to bend laterally, while preventing unwanted rotation of the complex.

**Conclusions:**

In conclusion, the MARPE may be beneficial for hyperdivergent patients, or those that have already experienced closure of the midpalatal suture, who require palatal expansion and would worsen from buccal tipping of the teeth or maxillary complex.

## Background

### Rapid palatal expansion

The prevalence of maxillary transverse deficiency is 8% to 23% in the deciduous and mixed dentitions and less than 10% in adult orthodontic patients [1–5]. While the cause of maxillary constriction is multifactorial [6], one way to alleviate this skeletal deficiency is through rapid palatal expansion (RPE).

RPE separates the two maxillary bones at the midpalatine suture [7,8]. During expansion, the force of the appliance counteracts the existing anatomical resistance from the dentoalveolus, midpalatal suture, zygomaxillary buttress, and circummaxillary sutures [9–15]. Chaconas and Caputo concluded that the major resistance to expansion forces was not the midpalatal suture but other articulations in the maxilla, such as the zygomatic and sphenoidal sutures [16]. Other RPE studies have proposed that during the opening of the midpalatal suture, the maxilla moves downward and forward [17], supporting the theories of disarticulation and separation of maxillary segments. However, conventional RPE provokes an orthodontic effect of buccal tipping and movement of the posterior teeth.

**Figure 1:**
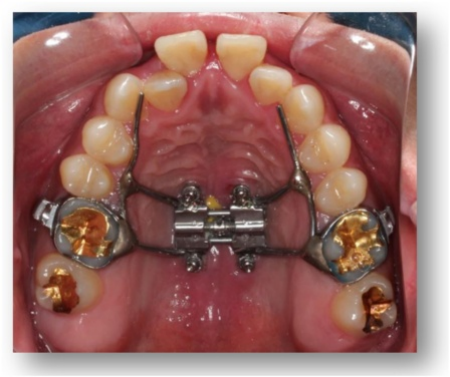
**MARPE.**

In recent years, a third palatal expansion design has been developed with a jackscrew attached to the palatal vault by a temporary anchorage device (Figure 1) [18]. This design is the micro-implant assisted rapid palatal expander (MARPE), used to combat undesired dental effects by achieving pure skeletal changes.

### MARPE

MARPE is a simple modification of a conventional RPE appliance. The main difference is the incorporation of micro-implants into the palatal jackscrew to ensure expansion of the underlying basal bone, minimizing dentoalveolar tipping and expansion. The literature shows a lack of knowledge and data regarding MARPE in the orthodontic community, yet many clinicians continue to utilize the device in practical or educational settings. Several case presentations have been published [18–21], but no studies demonstrate the influence of the MARPE on the cranium and surrounding circummaxillary sutures.

Tausche et al. reported that a MARPE is a viable expansion technique, allowing for the protection of teeth and preventing buccal tipping of the posterior dentoalveolar segment by 10° [18]. Nienkemper et al. reported that the mentioned side effects of RPE appliances can be minimized using a hybrid hyrax device that is connected to two orthodontic micro-implants in the anterior palate and is also attached to the first molars [22]. The disadvantages of MARPE are the difficulty in keeping the area clean, the invasiveness of the micro-implants, and the increased risk of infection.

### FEM

Finite element method (FEM) is an approximation method, replacing a complex structure with an assemblage of simple elements interconnected at points called nodes. These elements can be assembled to represent any shape or defined model [23]. Each element can be assigned with material properties that are determined by the clinical situation or model conditions, and forces are applied to simulate clinical loads. The experimental response to the applied forces or applied stress can then be visualized and calculated. FEM allows for detailed visualization of strength and stiffness where structures bend and twist, while indicating the distribution of displacements and stresses [24].

In this study, FEM was used to evaluate stress and strain within the craniofacial complex when transverse forces are independently applied to the palatal vault and maxillary teeth, effectively comparing conventional hyrax and MARPE expanders. With the inability to compose such studies in human clinical trials, this method is non-invasive and non-destructive. FEM allows for the examination of internal and external surfaces of the maxilla and surrounding structures, while also simulating changes to clinical orthodontic treatment. The simulated findings may be applied to the improvement of clinical protocols.

#### Hypotheses

The hypotheses of the study are as follows:

- Stress distribution from transverse forces applied to the dentition for conventional hyrax propagates beyond the dentoalveolar regions to the surrounding nasomaxillary complex.
- In contrast, the stress distribution from transverse forces applied to the palate for MARPE will cause resultant stresses that will be concentrated closer to the point of application.
- The transverse forces applied for the conventional hyrax will cause a greater horizontal rotation of the maxillary complex when compared to the MARPE.
- The patent model for both the conventional hyrax and MARPE will allow for greater propagation of the forces to the surrounding nasomaxillary complex.

## Methods

### Spiral CT

Computed tomography (CT) data was obtained from a 42-year-old male patient from the Ohio University Department of Biomedical Sciences. The spiral CT scanning was performed with the following parameters: 120 kV, 360 mA, matrix size of 512 × 512, and the slice thickness of 0.300 mm and voxel 0.463 × 0.463 × 0.300. The CT was used to generate the three-dimensional (3D) model for the finite element analysis (FEA) in the study. In addition, the 3D skull image was trimmed to exclude the mandible.

### Finite element analysis

The finite element solution is divided into three stages: preprocessing, solution, and postprocessing. The preprocessing stage consists of taking the 3D skull and developing an FEM model with assigned material properties. The solution stage is where boundary conditions are determined and forces are applied. Finally, postprocessing allows for analysis and viewing of the results.

#### Preprocessing

##### FEM model generation

**Figure 2:**
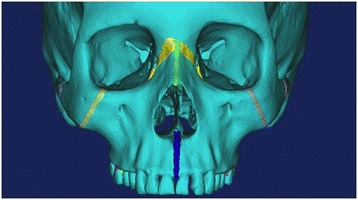
**3D Skull with masked sutures.**

The volumetric data from the CT scan was imported into Mimics 13.1 software (Materialise: Leuven, Belgium), and a 3D model of the patient's skull was generated. The 3D mask of the skull was then modified to create separate masks for the sutures that articulate with the maxilla. The following masks were created: zygomaticomaxillary (2), zygomaticotemporal (2), midpalatal, median nasal, and lateral nasal (2), and pterygomaxillary (2) (Figure 2). Studies have concluded that the average width of cranial sutures is unknown and considered highly variable and, however, are estimated to range between 1 and 2.5 mm [25]. The manually generated suture masks were 1.5–2 mm in width.

The next step was the development of the FEM mesh, where triangle reduction was completed with the removal of any misshapen or overlapping triangles.

##### Assignment of material properties

The mechanical properties assigned to the elements were isotropic (having a physical property that has the same value when measured in different directions) and linear-elastic (linear relationships between the components of stress and strain). The skull was assigned the Young's modulus and Poisson's ratio for compact bone, *E* = 1.37 × 10^3^ (kg/mm^2^) and *ν* = 0.3 [26,27]. For the patent FE model, the suture elements were assigned the values of connective tissue, *E* = 6.8 × 10^−2^ (kg/mm^2^) and *ν* = 0.49 [26,28,29].

### Solution

#### Boundary condition

Nodes along the foramen magnum were fully constrained by all degrees of freedom, zero rotation, and zero displacement [24].

#### Force application

**Figure 3:**
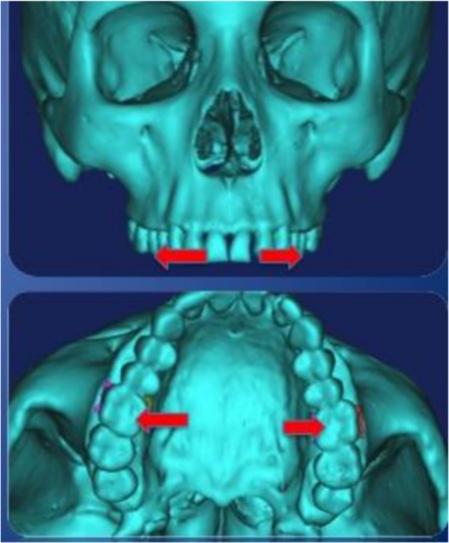
**Location of conventional hyrax force application.**

**Figure 4:**
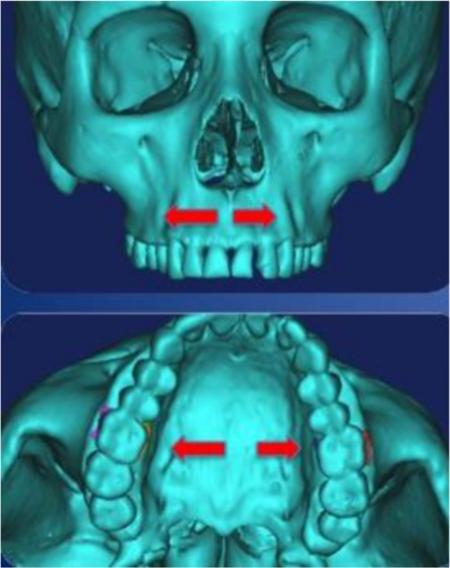
**Location of MARPE force application.**

Two sets of different locations for expansion forces were applied to mimic the conventional hyrax and the MARPE. For the conventional hyrax, the maxillary first molars are typically used for the attachment of the palatal expansion device. Equal forces were applied transversely along the *X*-axis to the lingual of the maxillary first molars at the center of the clinical crown (Figure 3). With the MARPE, palatal microscrews are inserted at the apex of the palate 3 mm lateral to the midpalatine suture. Equal forces were applied bilaterally along the *X*-axis at the point of insertion of the microscrews (Figure 4). Values of 800 g per side were applied for both conventional hyrax and MARPE [30].

The two sets of possible locations were separated into four total groups to be studied: group 1 was the patent model with the forces imitating the conventional hyrax, group 2 was the fused model with the forces imitating the conventional hyrax, group 3 was the patent model with the forces imitating the MARPE, and group 4 was the fused model with forces imitating the MARPE.

### Postprocessing

Utilizing ANSYS software, nodal and element solutions were plotted and areas of high stress concentrations were identified. Specific nodes on the skull were selected, and the stress values were exported to Microsoft Excel for analysis and graphing.

#### Nodal stress values

Surface nodes were selected to quantify the differences of first and third principle stress as well as von Mises stress. First principle stress measures tensile stress while the third principle stress measures compressive stress.

The von Mises stress is derived from the *distortion energy theory* and is a measurement to combine stresses on all three dimensions (first, second, and third principle stresses). The principal stresses are calculated at each point in the model through the cubic equation below:

$$ \sigma \left[\begin{array}{ccc}\hfill {\sigma}_{11}\hfill & \hfill {\sigma}_{12}\hfill & \hfill {\sigma}_{13}\hfill \\ {}\hfill {\sigma}_{21}\hfill & \hfill {\sigma}_{22}\hfill & \hfill {\sigma}_{23}\hfill \\ {}\hfill {\sigma}_{31}\hfill & \hfill {\sigma}_{31}\hfill & \hfill {\sigma}_{31}\hfill \end{array}\right]=\left[\begin{array}{ccc}\hfill {\sigma}_{x x}\hfill & \hfill {\sigma}_{x y}\hfill & \hfill {\sigma}_{x z}\hfill \\ {}\hfill {\sigma}_{y x}\hfill & \hfill {\sigma}_{y y}\hfill & \hfill {\sigma}_{y z}\hfill \\ {}\hfill {\sigma}_{z x}\hfill & \hfill {\sigma}_{z y}\hfill & \hfill {\sigma}_{z z}\hfill \end{array}\right]=\left[\begin{array}{ccc}\hfill {\sigma}_x\hfill & \hfill {\sigma}_{x y}\hfill & \hfill {\sigma}_{x z}\hfill \\ {}\hfill {\sigma}_{y x}\hfill & \hfill {\sigma}_y\hfill & \hfill {\sigma}_{y z}\hfill \\ {}\hfill {\sigma}_{z x}\hfill & \hfill {\sigma}_{z y}\hfill & \hfill {\sigma}_z\hfill \end{array}\right] $$

By expanding the determinant, an equation of the cubic order is determined, and three roots from this equation are derived as solution. The roots are then arranged in an order so as to get the most positive (tensile), most negative (compressive) values and are defined as *σ*1, *σ*3, and *σ*2 (first, third, and second principle stresses), respectively. With the first principle and third principle being the most positive and most negative values, the second principal stress is not of significance because the value is usually near zero.

Surface nodes were selected based on the areas of high stress observed visually. The groups of surface nodes selected were near the zygomaxillary suture (seven nodes), zygomaxillary buttress (seven nodes), lateral nasal suture (three nodes), buccal alveolar bone (three nodes), medial orbit (five nodes), lateral pterygoid plate (four nodes), medial pterygoid plate (four nodes), palatal shelf (nine nodes), and lingual alveolar bone (four nodes). Additionally, pieces of the skull were removed to reveal interior nodes where stresses were measured. The groups of nodes selected were near the lateral nasal wall (six nodes), roof of the nasal cavity (six nodes), internal key ridge (one node), lateral pterygoid plate (seven nodes), zygomaxillary suture (one node), and first molar (one node).

## Results

### Model comparison

#### Frontal view

**Figure 5:**
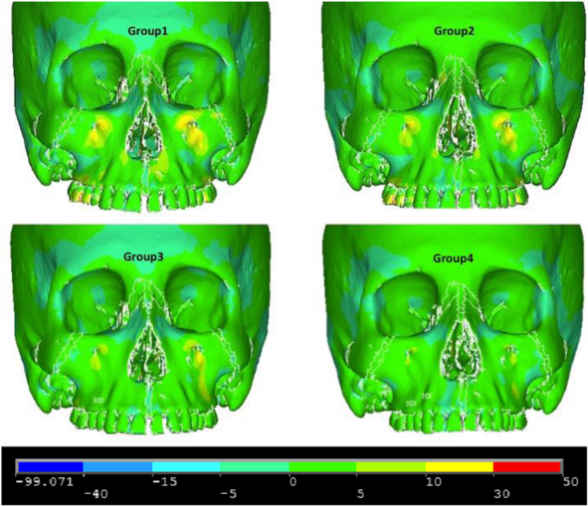
**First principle stress—frontal view.**

**Figure 6:**
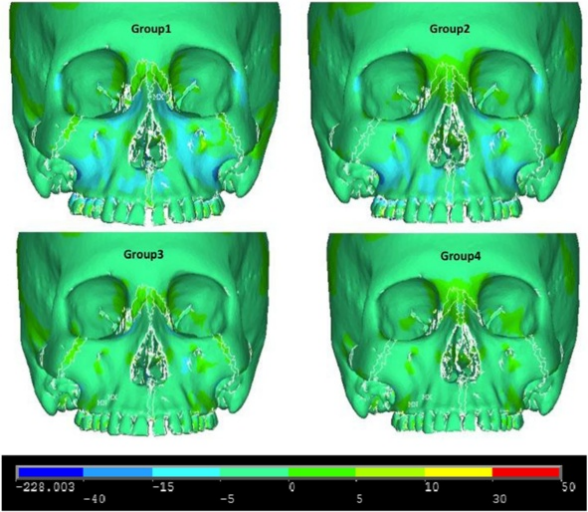
**Third principle stress—frontal view.**

**Figure 7:**
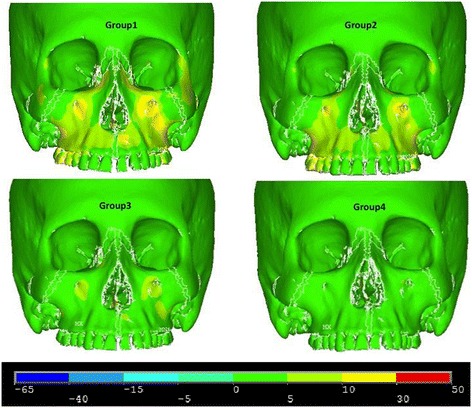
**von Mises stress—frontal view.**

Figures 5, 6 and 7 illustrate anterior views of groups 1–4 and the stress patterns resulting from force application.

Group 1 and group 2 exhibit similar stress patterns, whereas group 1 displays broadened tensile stresses near the anterior nasal aperture and the zygomaxillary suture adjacent to the infraorbital foramen (Figure 5). When comparing to group 1 and group 2, group 3 and group 4 demonstrate small overall stresses around the infraorbital forament but very little stress anywhere else in the anterior view (Figure 7). Group 3 portrayed a slightly broadened overall stress around the infraorbital foramen when compared to group 4 (Figure 7). Also, group 1 displays more widespread compressive stresses that extend up to the inferior border of the lateral nasal suture (Figure 6). Lastly, group 1 presents with a small amount of overall stress near the lateral border of the orbit and zygoma (Figure 7).

### Lateral view

**Figure 8:**
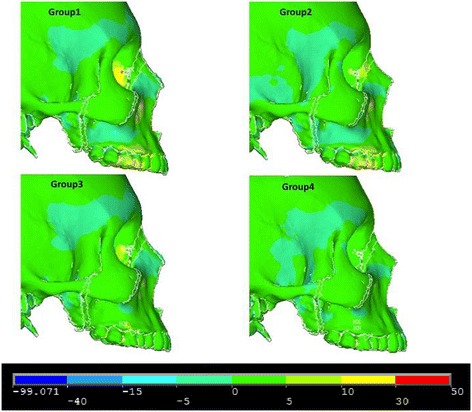
**First principle stress—lateral view.**

**Figure 9:**
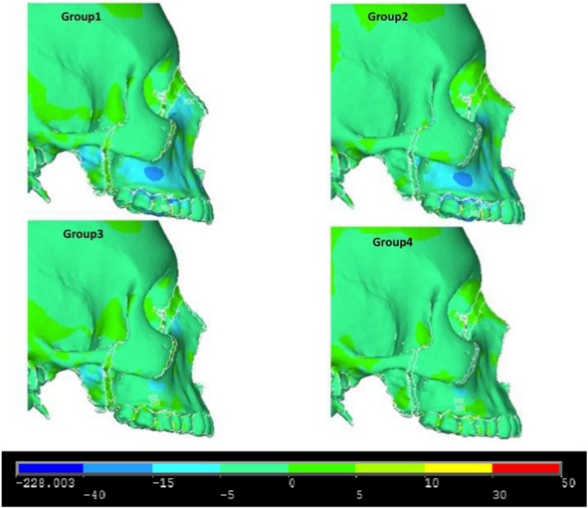
**Third principle stress—lateral view.**

**Figure 10:**
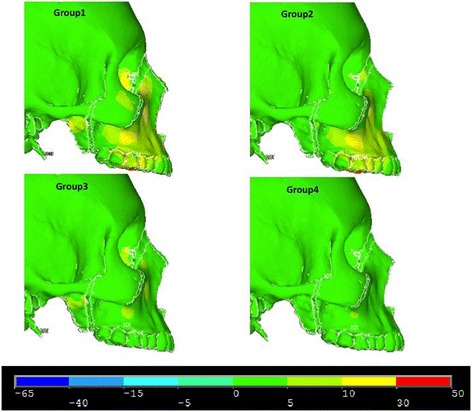
**von Mises stress—lateral view.**

Figures 8, 9 and 10 illustrate anterior views of groups 1–4 and the stress patterns resulting from force application.

In the lateral view, significant compressive stresses are seen at the zygomaxillary buttress and molars for groups 1 and 2 (Figure 9). Group 1 and group 3 reveal compressive stress at the superior portion of the lateral pterygoid plates (Figure 9). Both group 1 and group 2 demonstrate tensile stress around the buccal plates of the molars and premolars (Figure 8). Similar to the frontal view, group 1 and group 2 showcase similar overall stress patterns; however, group 1 exhibits significantly greater stresses around the medial orbit and lateral pterygoid plate (Figure 10). Additionally, there is mild stress shown around the lateral border of the zygoma (Figure 10). In comparison to group 2, group 3 shows greater overall stress around the lateral pterygoid plate as well as the medial orbit (Figure 10). By and large, group 3 and group 4 display minimal stress when compared to groups 1 and 2 (Figure 10).

### Inferior view

**Figure 11:**
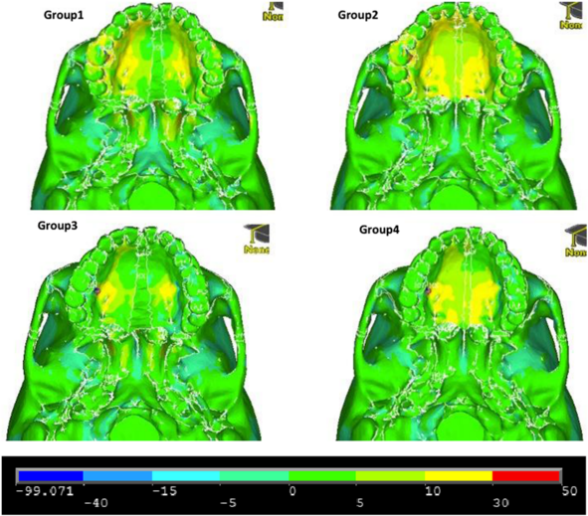
**First principle stress—occlusal view.**

**Figure 12:**
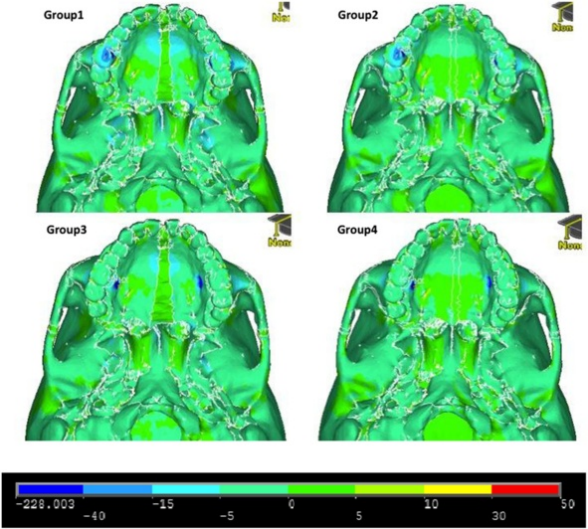
**Third principle stress—occlusal view.**

**Figure 13:**
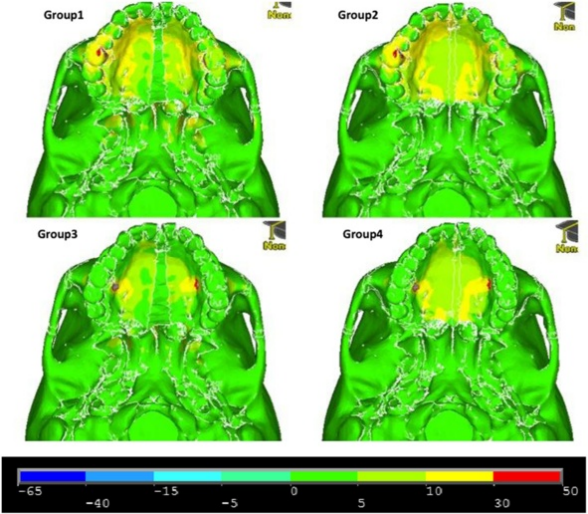
**von Mises stress—occlusal view.**

Figures 11, 12 and 13 illustrate anterior views of groups 1–4 and the stress patterns resulting from force application.

Group 1 exhibits tensile stresses around the palatal vault of the molars and premolars, extending anteriorly around the lingual bone of the central incisors. Significant stress is also seen around the medial and lateral pterygoid plates (Figure 11). In comparison, group 2 demonstrates a broader tensile stress distribution around the whole palate, with high stress at the distal aspect of the hard palate (Figure 11). Group 3 and group 4 reveal high tensile stresses around the implant sites; however, group 4 is more widespread, extending to the distal and anterior parts of the hard palate (Figure 11). Similar to group 1, group 3 displays high tensile stress around the lateral and medial pterygoid plates (Figure 11). Not surprisingly, high compressive stresses are seen around the points of force application (Figure 12). Group 1 and group 3 also display moderate compressive stresses around the lateral pterygoid plates (Figure 12).

#### Individual group stress values

*Group 1*. Patent model with the forces directed transversely between the UR6-UL6 (conventional hyrax with sutures).

**Figure 14:**
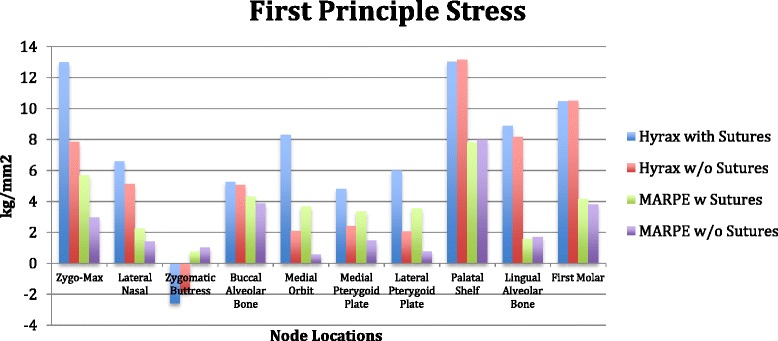
**First principle stress.**

**Figure 15:**
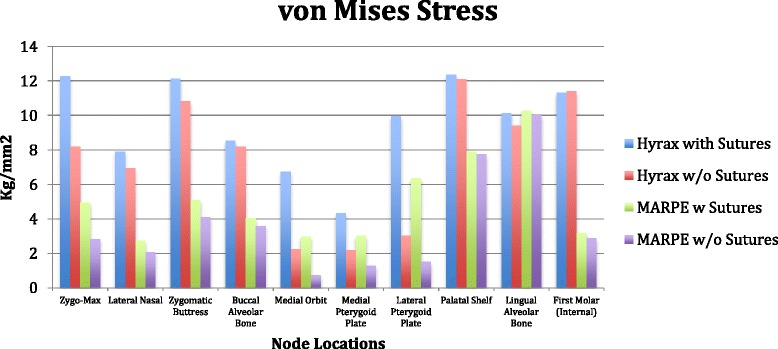
**von Mises stress.**

The first principle stress distribution plots show high tensile stresses near the zygomaxillary sutures radiating from the inferior part of the orbit down to the zygomaxillary buttress. Seven nodes were selected adjacent the zygomaxillary suture, and the average of those values was 12.98 kg/mm^2^ (Figure 14). Two additional areas of high tensile stress were the palatal shelf and the internal node of the first molar. The palatal shelf nodes were measured from the palatal vault adjacent to the upper right lateral incisors back to the upper right third molar (Figure 15). These palatal nodes averaged 13.01 kg/mm^2^. One node was analyzed internally for the first molar approximating the root furcation and measured 10.47 kg/mm^2^. Moderate stresses were observed in three areas: the lingual alveolar bone, medial orbit, and the lateral nasal suture. The averages of these locations were 8.88 kg/mm^2^, 8.30 kg/mm^2^, and 6.57 kg/mm^2^, respectively (Figure 14). Additional noteworthy tensile stresses were found at the buccal alveolar bone, medial pterygoid plate, and lateral pterygoid plate, which measured 5.24 kg/mm^2^, 4.81 kg/mm^2^, and 6.00 kg/mm^2^, respectively (Figure 14).

**Figure 16:**
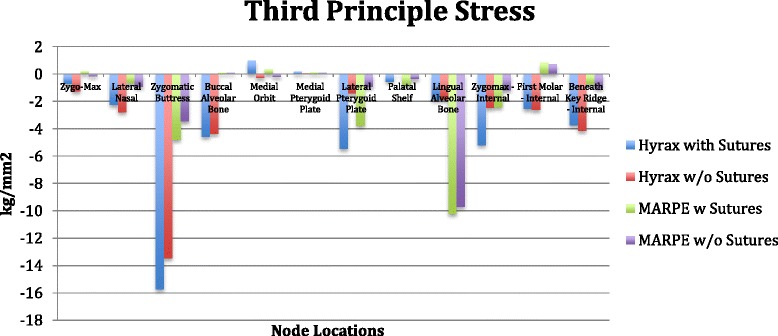
**Third principle stress.**

The third principle stress distribution plots display high compressive stresses around the zygomaxillary buttress nodes and averaged −15.73 kg/mm^2^ (Figure 16). Moderate compressive stress was found at the lateral pterygoid plate, buccal alveolar bone, and zygomaxillary internal node, with the averages from those locations being −5.48, −4.59, and −5.2 kg/mm^2^, respectively (Figure 16).

von Mises stress, which is a combination of all stresses in three dimensions, exhibited high values in the areas of the zygomaxillary sutures (12.28 kg/mm^2^), zygomaxillary buttress (12.14 kg/mm^2^), lateral pterygoid plate (9.96 kg/mm^2^), palatal shelf (12.37 kg/mm^2^), and lingual alveolar bone (10.13 kg/mm^2^) (Figure 15). Moderate stresses were shown at the lateral nasal suture (7.9 kg/mm^2^), buccal alveolar bone (8.54 kg/mm^2^), and medial orbit (6.75 kg/mm^2^) (Figure 15).

*Group 2.* Fused model with the forces directed transversely between the UR6-UL6 (conventional hyrax without sutures).

The first principle stress distribution plot exhibited high tensile stresses on the nodes located in the palatal shelf (13.15 kg/mm^2^) and the first molar internal node (10.5 kg/mm^2^) (Figure 14). Moderate tensile stress is seen at the zygomaxillary sutures (7.84 kg/mm^2^), lingual alveolar bone (8.15 kg/mm^2^), lateral nasal suture (5.11 kg/mm^2^), and buccal alveolar bone (5.05 kg/mm^2^) (Figure 14).

The third principle stress distribution plots presented with high compression stress levels around the zygomaxillary buttress (−13.47 kg/mm^2^) (Figure 16). In addition, there was mild compressive stress demonstrated at buccal alveolar bone (−4.37 kg/mm^2^) and lateral nasal suture (−2.8 kg/mm^2^) (Figure 16).

The von Mises stresses were significantly elevated in the nodes of the first molar (11.42 kg/mm^2^), zygomatic buttress (10.83 kg/mm^2^), and palatal shelf (12.09 kg/mm^2^) (Figure 15). Moderate stress is seen in the nodes of the zygomaxillary suture (8.19 kg/mm^2^), lingual alveolar bone (9.41 kg/mm^2^), buccal alveolar bone (8.18 kg/mm^2^), and lateral nasal suture (6.93 kg/mm^2^) (Figure 15).

*Group 3.* Patent model with the forces directed transversely toward the UR6-UL6 adjacent to the midpalatal suture (MARPE with sutures).

The first principle stress distribution plot exhibited only moderate tensile stresses on the nodes located in the palatal shelf (7.79 kg/mm^2^) and the zygomaxillary suture (5.69 kg/mm^2^) (Figure 14). Mild tensile stresses were seen at the buccal alveolar bone (4.32 kg/mm^2^), medial orbit (3.66 kg/mm^2^), lateral pterygoid (3.54 kg/mm^2^), and medial pterygoid (3.36 kg/mm^2^) (Figure 14).

The third principle stress distribution plots presented with high compression stress levels at the lingual alveolar bone (−10.22 kg/mm^2^) (Figure 16). Also, there was mild compressive stress demonstrated at zygomaxillary buttress (−4.81 kg/mm^2^) and lateral pterygoid (−3.78 kg/mm^2^) (Figure 16).

The von Mises stresses were prominent in the nodes of the lingual alveolar bone (10.29 kg/mm^2^) and moderate in the nodes of the palatal shelf (7.90 kg/mm^2^) and lateral pterygoid plate (6.36 kg/mm^2^) (Figure 15). Mild stresses were also evident in the nodes of the zygomaxillary suture (4.95 kg/mm^2^), zygomaxillary buttress (5.09 kg/mm^2^), buccal alveolar bone (4.06 kg/mm^2^), medial orbit (2.98 kg/mm^2^), and medial pterygoid plate (3.03 kg/mm^2^) (Figure 15).

*Group 4.* Fused model with the forces directed transversely toward the UR6-UL6 adjacent to the midpalatal suture (MARPE without sutures).

The first principle stress distribution plot measured only moderate tensile stresses on the nodes located in the palatal shelf (7.97 kg/mm^2^) (Figure 14). Mild tensile stresses were seen at the buccal alveolar bone (3.87 kg/mm^2^), zygomaxillary suture (2.95 kg/mm^2^), and internal node of the first molar (3.79 kg/mm^2^) (Figure 14).

The third principle stress distribution plots demonstrated high compression stress levels at the lingual alveolar bone (−9.7 kg/mm^2^) and only mild compressive stress at zygomaxillary buttress (−3.47 kg/mm^2^) (Figure 16).

The von Mises stresses were high in the nodes of the lingual alveolar bone (10.01 kg/mm^2^) and moderate in the nodes of the palatal shelf (7.74 kg/mm^2^) (Figure 15). Mild stresses were measured in the nodes of the zygomaxillary suture (2.84 kg/mm^2^), zygomaxillary buttress (4.09 kg/mm^2^), buccal alveolar bone (3.6 kg/mm^2^), and lateral nasal suture (2.08 kg/mm^2^) (Figure 15).

## Discussion

New appliances, such as the MARPE have been tested in orthodontic patients with hopes of avoiding the unwanted side effects of traditional RPE. While the MARPE has shown evidence of clinical success [18–21], most are limited in the precise evaluation of the biomechanical effect of orthopedic forces, and it is difficult to suggest exactly what is taking place physiologically.

Recent studies have demonstrated that FEM is a viable method to study stress, strain, and force distributions when evaluating orthodontic problems, specifically transverse deficiencies [26,31–33]. In a non-invasive way, FEM makes it possible to compare the effects of conventional hyrax and MARPE expansion forces on the craniofacial complex.

### Model generation

The FEM model of the cranium was generated using Mimics software and consisted of 91,933 nodes and 344,451 elements. Interestingly, previously published FEM RPE studies have generated models that are isotropic with only a midpalatal suture. The midpalatal suture certainly is important; however, skeletal resistance to maxillary expansion will emanate primary to the three maxillary buttresses: pterygomaxillary, zygomaticomaxillary, and nasomaxillary [26,34,35]. In order to achieve an accurate representation of the nasomaxillary complex, the circummaxillary sutures are essential. In our study, the following sutures were included: zygomaticomaxillary, zygomaticotemporal, midpalatal, median nasal, lateral nasal, and pterygomaxillary. Similar to previously published studies that utilized the midpalatal suture and periodontal ligament (PDL) [26,28], our study applied the material property of connective tissue for all sutures. Pirelli et al. demonstrated that the periodontal ligament and the midpalatal suture have similar histological characteristics to that of connective tissue [29].

The periodontal ligament around the maxillary first molars was not modeled, but this omission has minimal or no effect on the postprocessing results of the conventional palatal expansion. In addition, it is believed that the thin PDL would have an insignificant affect on the large forces applied.

### Force application

From the two models, two sets of locations for force application were evaluated, totaling four simulations. For group 1 and group 2, the conventional hyrax was simulated, where transverse expansion forces of 800 g were applied to the lingual of the maxillary first molars. Group 1 was the model with sutures, and group 2 was without. For these two groups, 5 mm of expansion from lingual cusp to lingual cusp of the maxillary first molars was achieved, which helped to validate the model and force level. It was assumed that 5 mm of expansion would be enough to correct most crossbites in adolescents and adults. An expansion force of 800 g was determined to be reasonable for our study because previous reports have demonstrated that approximately 700–900 g of force is applied to the teeth during maxillary expansion [30]. Group 3 and group 4 illustrated the MARPE expander, with the simulations representing patent and fused sutures, respectively. Forces of 800 g were directed transversely in the same lateral direction as the conventional hyrax, but at the apex of the palate, 3 mm lateral to the midpataline suture. Clinically, with the MARPE design, the jackscrew is anchored to the palate with micro-implants 2–3 mm lateral to the midpalatine suture at the level of the maxillary first molars.

The distributions of forces follow very distinct trajectories in the first two groups and are dispersed from the site of application. In the conventional hyrax groups, the forces not only affect the structures around the point of application but also the distant structures in the midface and cranial base. During conventional palatal expansion, the forces must overcome the resistance of stress-dissipating structural pillars, referred to as maxillary buttresses [36]. From the first principle, third principle, and von Mises stress distribution plots of the conventional hyrax expansion groups, it is evident that the maxillary buttresses are the main areas of resistance during heavy orthopedic expansion. With the forces on the maxillary molars, the stress radiates to the three main buttresses of the midface-cranial complex: the nasomaxillary, the zygomaticomaxillary, and the pterygomaxillary. The resulting FEM stress distributions are similar to previous studies and demonstrate that stress trajectories follow the structure of the maxillary buttresses [26,35,36]. The correlation of the findings validates our FEM model.

Previous studies have identified the center of resistance of the maxilla in both the sagittal and frontal views. From a frontal view, the centers are referred to as an intersection of two axis: the first through the crista galli and the second through the most inferior points of the zygomaticomaxillary sutures bilaterally. The center of resistance is located at the perpendicular intersection of these two axes. From a lateral view, the center of resistance is located along a line passing through the distal contact of the maxillary first molar to the functional plane and then taking half of the distance from the functional plane to the inferior border of the orbit [37]. When the jackscrew is activated during conventional expansion, the force on the maxillary teeth creates equivalent moments at the centers of resistance of each maxillary half. Biomechanically, there are two centers of rotation, one at the frontonasal suture, and the other is distal to the midpalatal suture in the area of the third molar [38]. The fulcrum of the maxillary rotation is at the frontomaxillary suture, and as a result, there exists a triangular intermaxillary diastasis with its base at the level of the upper incisors and the apex at the level of the nasal cavity [39]. In a previous study, patients who underwent palatal expansion reported symptoms of heavy pressure at the bridge of the nose and under the eyes [30]. These clinical symptoms correlate well with the location of the maxillary fulcrum of rotation and the group 1 and group 3 stress patterns in the frontonasal, frontomaxillary, and nasomaxillary sutures. These two groups showed significant overall stresses near the nose and medial orbit, with group 1 showing the greatest effect. The suture properties of these two groups allowed for a larger flexing of the maxillary complex and a greater stress expression near the nose. Interestingly, even in the fused conventional hyrax model (group 2), mild overall stress was seen around the medial orbit.

As was stated, the furthest point from the maxillary fulcrum of rotation is at the level of the dentoalveolus. With the location of the adjoining zygoma, when force is placed similar to a conventional hyrax, compressive stress should be seen in the area of the zygomaticomaxillary buttress. Naturally, in both conventional hyrax simulations, these areas manifested high compressive stress levels.

From a clinical perspective, placement of the jackscrew should be as close to the center of resistance as possible to effect a more translatory movement of the maxillary halves. With a conventional hyrax, it is impossible to direct the force from the jackscrew through the center of resistance to produce pure bodily movement. It is believed that with a more rigid expansion appliance, the center of rotation will move superiorly and posteriorly [38].

In the MARPE simulation groups, the point of force application is closer to maxillary fulcrum of rotation as well as the center of resistance. According to Braun et al., this change in force application will result in a more linear separation of the maxillary halves [38]. The stress distribution shown in group 3 and group 4 demonstrate this phenomenon, as the stress is more centrally concentrated around the site of application. The MARPE groups display significantly less stress around the three main buttresses. In addition, the point of force application is at the peak of the palate, which prevents the unwanted dental movement and the bending of the maxillary complex that can often take place in conventional hyrax expansion. Not surprisingly, no stress is seen in the area of the first molars.

It is of interest to note the significant differences in the patent versus fused models. The fused model more accurately approximates the response of an adult maxilla. With the interlocking of the midpalatal and the surrounding maxillary sutures, minimal flexion is seen, and less stress is dissipated to the contiguous nasomaxillary complex. Significantly, greater stress is seen at the point of force application, specifically around the lingual of the first molars in the conventional hyrax group, and the implant site of the MARPE group. If the midpalatal suture is completely fused, stress will not be distributed to the surrounding structures because the palate is able to withstand the lateral force applied by the MARPE. Conversely, the patent groups demonstrated a more widespread stress pattern, which can be attributed to the flexible nature of the nasomaxillary sutures. However, the expansion may never happen if the midpalatal suture is completely fused. The stress is not felt in the surrounding structures because the fused palate is able to withstand the lateral force applied by the MARPE. Group 1 and group 3 showed stress along all three maxillary buttresses; however, the conventional hyrax group revealed significantly higher stress in all three areas.

### Study limitations

The results of this study are based on a model that was generated from a CT scan of a 42-year-old male patient. While the study provided a greater understanding of MiRPE stress distribution within the craniofacial complex, the results are only valid for patients with comparable craniofacial structure. Sutures were incorporated into the model to mimic an adolescent, but the general size and shape of the model is more similar to that of an adult male. Additionally, FEM is only as good as the model upon which it is based. In this study, it was assumed that the elements have isotropic properties and are time-independent, yet in an actual skull, the bone is more anisotropic with a time-dependent component. In essence, FEM does not demonstrate longitudinal effects, only a particular instance in time. As such, future studies that allow a time-dependent change in the model should be explored.

Also, the properties assigned to the cranial bones were isotropic, meaning the model material's physical property has the same value when measured in different directions. In our model, no distinction was made between cortical and cancellous bone, and all hard tissues were given the mechanical property of cortical bone. When a solid model is developed in Mimics from a CT scan, no distinction is made to differentiate between bone types. For future studies using the current software, the variations between the bone types would have to be manually inputted, which at this point would be estimation. Unfortunately, this study also involves several approximations in the material properties of soft tissues, with sutures assigned the mechanical property of connective tissue. Also, the suture configurations were estimated due to difficulty in extracting this information from the scanned image. Variations in teeth, enamel, dentin, pulp, and PDL structures were not considered in this study. Future refining of these anatomical structures using a more sophisticated computer program can seek to address this.

### Clinical applications

The results of this FEM study demonstrate that by altering the location of the expansion force, the stress distribution on craniofacial complex is changed. By placing the jackscrew closer to the center of resistance, a more horizontal translation of the maxilla will take place, with less resultant complex tipping. Also, with the MARPE appliance anchored to the palate rather than the teeth, less dental tipping will take place, which will allow for better vertical control.

Questions have been raised about non-surgical maxillary expansion in adults, and the consensus is that once patients are out of their teens, conventional expansion is no longer feasible. Interestingly, this study demonstrated that with the fused model, maxillary expansion with the MARPE may be possible if the expansion force can split the suture. Applying a significantly higher level of force may be possible without adversely affecting the surrounding structure. Recently, clinical studies have verified the findings of our study, and we discovered that micro-implant assisted palatal expansion is possible in adults.

## Conclusions

The conclusions are as follows:

- FEM is a valid method for comparing the effects of maxillary expansion appliances on the craniofacial complex. FEM modeling software now allows for the incorporation of sutures, for a more accurate and convincing model.
- Stress distribution from conventional expansion was distributed along the three maxillary buttresses: the zygomaticomaxillary, the nasomaxillary, and the pterygomaxillary. In comparison, stress distribution from the MARPE showed less propagation to the buttresses and adjacent locations in the maxillary complex.
- By placing expansion forces closer to the maxilla's center of resistance, less tipping occurs with a more lateral translation of the complex. Further clinical investigation is necessary, but this study suggests that MARPE can be beneficial in patients with sutures that are fused. Lastly, MARPE is also beneficial in young dolichofacial patients by helping to prevent bone bending and dental tipping.
